# Characterization of African Swine Fever Virus Caucasus Isolate in European Wild Boars

**DOI:** 10.3201/eid1712.110430

**Published:** 2011-12

**Authors:** Claudia Gabriel, Sandra Blome, Alexander Malogolovkin, Stanislav Parilov, Denis Kolbasov, Jens P. Teifke, Martin Beer

**Affiliations:** Friedrich-Loeffler-Institut, Greifswald–Insel Riems, Germany (C. Gabriel, S. Blome, J.P. Teifke, M. Beer);; National Research Institute for Veterinary Virology and Microbiology, Pokrov, Russia (A. Malogolovkin, S. Parilov, D. Kolbasov)

**Keywords:** viruses, African swine fever virus, ASF, vector-borne infections, arboviruses, Caucasus isolate, wild boar, experimental characterization, Europe, arthropod-borne virus

## Abstract

Since 2007, African swine fever has spread from the Caucasus region. To learn more about the dynamics of the disease in wild boars (*Sus scrofa*), we conducted experiments by using European wild boars. We found high virulence of Caucasus isolates limited potential for establishment of endemicity.

African swine fever (ASF) is one of the most serious diseases affecting pigs (*1*). The causative agent, *African swine fever virus* (ASFV), is a complex DNA virus of the genus *Asfivirus* within the *Asfarviridae* family. Because of its ability to replicate in *Ornithodorus* ticks, ASFV can be classified as arthropod-borne virus (*2*). In domestic pigs, ASFV can cause a wide range of clinical signs, including hemorrhagic syndromes with high lethality. Little is known about ASF in European wild boars, although indications exist that the animals are highly susceptible (*3*).

In 2007, ASF affecting domestic pigs and wild boars was reported in the Caucasus region. The virus strain involved was related to isolates of genotype II, which are circulating in Mozambique, Madagascar, and Zambia (*4*). Especially in Russia, ASF recurs and shows a clear tendency to move northward (*5*). This unresolved situation increases the risk of introducing the virus into virus-free areas, and the involvement of wild boar, raises special concerns. As seen with classical swine fever, the growing population of wild boars is problematic for animal disease control, particularly if the infection reaches endemicity (*6*). Therefore, knowledge about disease dynamics is vital for risk assessment and strategy design, particularly because no vaccine against ASF is available.

Therefore, animal experiments were carried out at the Friedrich-Loeffler-Institut (Greifswald–Insel Riems, Germany), and the National Research Institute for Veterinary Virology and Microbiology (NRIVVaMR, Pokrov, Russia). The aim was to define clinical signs, disease dynamics, and postmortem lesions in wild boars after intramuscular and oral infection with ASFV Caucasus isolates.

## The Study

The study comprised 2 experimental parts: 1) oral infection conducted at the Friedrich-Loeffler-Institut and 2) intramuscular infection at NRIVVaMR. For oral infection, we used a 2008 isolate from Armenia. The experiment was conducted by using 6 wild boar piglets 9 weeks of age. Three domestic pigs were used as contact controls and were handled in the same manner as the wild boar piglets. The animals were kept under high-containment conditions. After acclimatization, the wild boars were infected orally with 2 mL of a spleen suspension containing 10^6^ median tissue culture infectious dose ASFV/mL. Two days after infection, 3 domestic weaner pigs were added to the pen with the wild boar piglets. Starting from the day of infection, rectal temperature and clinical signs were recorded. Oral and fecal swabs were collected from the wild boars at 0, 1, 2, 3, 5, 6, and 7 days postinfection (dpi). In addition, blood samples were taken at 0, 2, 5, 6, and 7 dpi. Blood from the domestic pigs was sampled at 0, 6, 9, and 13 dpi. Necropsy was performed on all animals.

For real-time quantitative PCR (qPCR), viral DNA was extracted by using manual and automated extraction methods according to manufacturer instructions. Subsequently, qPCR was performed according to the protocol published by King et al. (*7*) with slight modifications by using an Mx3005P PCR Cycler (Stratagene, La Jolla, CA, USA).

For intramuscular infection, 4 wild boars 9 months of age were brought to the containment stables of the NRIVVaMR. One animal was inoculated intramuscularly with 1,000 hemadsorbing units 50% of a 2009 virus isolate from the Chechen Republic, which is identical to the isolate used in the oral trial in all genome fragments routinely sequenced. The remaining animals were housed together with the infected animal as contact controls.

Clinical signs of infection were recorded every day. Samples of visceral organs, skin, and hair were taken during necropsy and subjected to qPCR. Isolation of viral DNA was performed by using an in-house kit based on the modified method published by Boom et al. (*8*). The qPCR for ASFV detection was carried out according to the protocol published by King et al. (*7*) with a Rotorgene 6000 instrument (Corbett Research, Sydney, Queensland, Australia).

**Figure 1:**
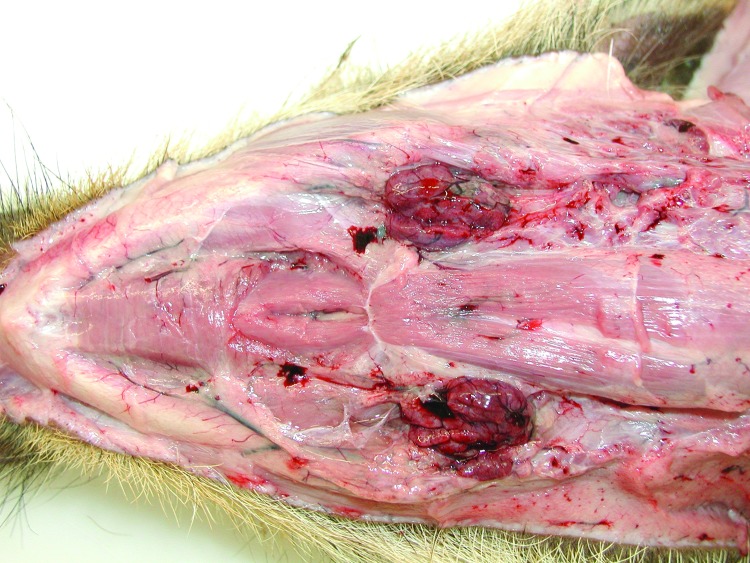
Ventral view of the head showing pathologic signs in a wild boar piglet after oral inoculation with 10^6^ median tissue culture infectious dose of an African swine fever virus isolate from Armenia (experiment at the Friedrich-Loeffler-Institut). Note edematously enlarged and hemorrhagic mandibular lymph nodes. The animal died on day 7 postinfection.

**Figure 2:**
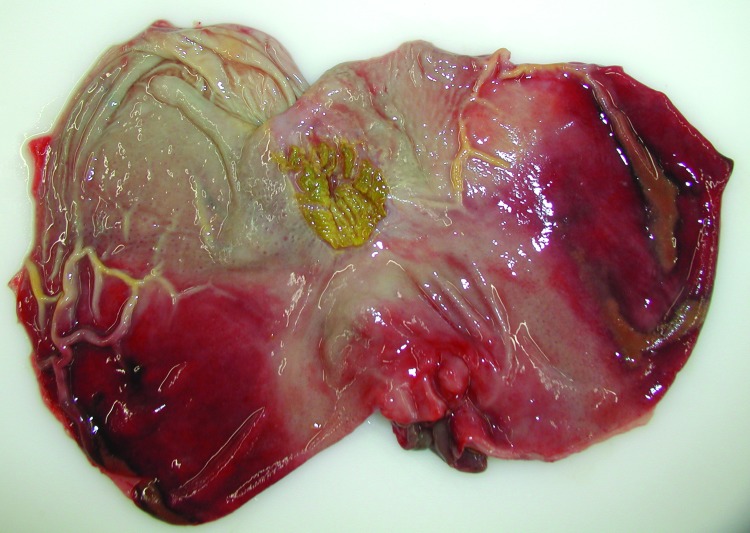
View of the mucosal surface of the dissected stomach showing representative gross lesions after oral inoculation of a wild boar with 10^6^ median tissue culture infectious dose of an African swine fever virus isolate from Armenia (experiment at the Friedrich-Loeffler-Institut). The image illustrates acute gastritis; note diffuse mucosal hemorrhages affecting a large part of the mucosa. The animal died on day 7 postinfection.

After oral infection, an acute fatal course of the disease developed in all wild boar piglets, and they died within 7 days. Apart from severe depression, slight diarrhea, and reduced feed intake, only high fever was observed starting 3–4 dpi. During postmortem examinations, enlarged and hemorrhagic lymph nodes (Figure 1) and hemorrhagic gastritis (Figure 2) were observed. Acute fatal ASF developed in 2 of the domestic pigs 11–12 dpi of the wild boars. These animals died 1 week later, showing severe but unspecific signs. One domestic pig became infected later. It only showed fever at 20 dpi and was euthanized on day 25. Infection of this animal was clearly linked to contact with blood from a moribund pen mate.

During the clinical phase of the disease, qPCR was positive for all blood samples with first positive results 2 dpi. Oropharyngeal and fecal swabs were positive mainly on days 6 and 7. An overview of the qPCR results is presented in Table 1.

**Table 1:** Real-time PCR results of blood and swab samples after oral infection in study of African swine fever virus in European wild boars*

Animal, sample source	Days postinfection of wild boar										
	0	1	2	3	5	6	7	9	13	17	20
Wild boar 1											
Blood	No C_t_	ND	No C_t_	ND	23						
Oropharyngeal swab	No C_t_	No C_t_	No C_t_	No C_t_	No C_t_						
Fecal swab	No C_t_	No C_t_	No C_t_	No C_t_	No C_t_						
Wild boar 2											
Blood	No C_t_	ND	No C_t_	ND	22	20	24				
Oropharyngeal swab	No C_t_	No C_t_	No C_t_	No C_t_	37	37	37				
Fecal swab	No C_t_	No C_t_	No C_t_	No C_t_	No C_t_	38	No C_t_				
Wild boar 3											
Blood	No C_t_	ND	No C_t_	ND	28	22	23				
Oropharyngeal swab	No C_t_	No C_t_	No C_t_	No C_t_	No C_t_	38	34				
Fecal swab	No C_t_	No C_t_	No C_t_	No C_t_	37	34	33				
Wild boar 4											
Blood	No C_t_	ND	No C_t_	ND	25	26	26				
Oropharyngeal swab	No C_t_	No C_t_	No C_t_	37	No C_t_	34	37				
Fecal swab	No C_t_	No C_t_	No C_t_	No C_t_	30	29	33				
Wild boar 5											
Blood	No C_t_	ND	39	ND	25	23					
Oropharyngeal swab	No C_t_	No C_t_	No C_t_	No C_t_	39	35					
Fecal swab	No C_t_	No C_t_	No C_t_	No C_t_	No C_t_	29					
Wild boar 6											
Blood	No C_t_	ND	No C_t_	ND	23	24					
Oropharyngeal swab	No C_t_	No C_t_	No C_t_	37	No C_t_	34					
Fecal swab	No C_t_	No C_t_	No C_t_	No C_t_	35	32					
Domestic pig 1, blood	No C_t_	ND	ND	ND	ND	39	ND	No C_t_	21	20	
Domestic pig 2, blood	No C_t_	ND	ND	ND	ND	No C_t_	ND	No C_t_	23	ND	
Domestic pig 3, blood	No C_t_	ND	ND	ND	ND	No C_t_	ND	No C_t_	No C_t_	ND	29†

*C_t_, cycle threshold; ND, not done because of missing samples. Numbers indicate C_t_ values. †Serum sample instead of whole blood sample was used.

On the third day after intramuscular inoculation, the infected wild boar showed depression, inappetence, and increased respiratory frequency. It died at 5 dpi showing hemorrhagic nasal discharge. The 3 contact animals showed similar symptoms at 8 dpi of the intramuscularly infected wild boar and died 2 days later. Postmortem examinations showed hemorrhages in multiple edematously enlarged lymph nodes, most prominent pulmonary hyperemia and alveolar edema, hyperplasia of the mesenteric lymph nodes, and acute gastritis with hemorrhages. Skin lesions were not present.

ASF genome was detected in the samples of visceral organs and lymph nodes of all animals. In samples of skin and kidneys, viral DNA was detected only in the infected animal. Results of qPCR are presented in the Table 2.

**Table 2:** Real-time PCR results of organ samples taken after intramuscular infection in a study of African swine fever virus in European wild boars*

Wild boar	Lung	Heart	Spleen	Lymph nodes
1	19	19	18	19
2	20	20	19	20
3	21	21	20	20
4	20	21	19	20

*Cycle threshold values indicated.

## Conclusions

Knowledge about disease dynamics in domestic pigs and wild boars is a prerequisite for risk assessment and prevention strategy design. Unfortunately, wild boar data are scarce. To contribute to this information, animal trials were conducted for an experimental characterization of recent Caucasian ASFV isolates in wild boars.

We concluded that the Caucasian isolates are highly virulent in wild boars. Both oral and intramuscular infection resulted in 100% lethality.

PCR results showed that the ASFV genome is easily detected in blood and organ samples of diseased animals. Swab samples were positive in the clinical phase of infection but showed much lower genome loads. Shedding of ASFV through nasal discharge or feces, and thus overall contagiousness, seems to be limited.

Transmission to domestic pigs was delayed in comparison to transmission to wild boars. The most likely reason for this difference seems to be contact with blood. Although this factor could be observed most certainly for the contact wild boars, domestic pigs had only limited contact with blood.

On the basis of these data, it seems unlikely the Caucasian isolates have the potential to become endemic in European wild boar populations without a distinct change in virulence. So far no indications exist that the virulence of ASFV is changing in affected regions in Russia.

A risk factor for disease control could be the involvement of tick vectors. Until now, no indications exist that ticks are involved in ASFV outbreaks in the Caucasus region and Russia. Moreover, it has to be kept in mind that the wild boar’s way of life does not facilitate contact with soft ticks. Nevertheless, this possibility was not examined during this study and needs further investigation.
